# Esmarch’s Operation for Closed Jaw

**Published:** 1877-05

**Authors:** 


Esmarch's Operation for Closed Jaw.—Mr. Annandale relates, in the *Edinburgh Medical Journal*, a case in which, after necrosis of the upper jaw, permanent closure of the jaws took place. This was remedied by excising a portion of the entire thickness of the lower jaw in front of the cicatrix, and about an inch to the left of the symphisis. By so doing, a false joint ultimately resulted at the point of excision, and the jaws were capable of being separated to the extent of three-quarters of an inch, whereas, formerly they could only be separated the eighth of an inch.—*The Doctor.*

---

Prof. Francis G. Smith has resigned the chair of Institutes of Medicine, in the University of Pennsylvania, (Med. Dept.) on account of ill-health. It is reported that other resignations in the same school are imminent.—*Hospital Gazette.*

